# Changes in cortical cytoskeletal and extracellular matrix gene expression in prostate cancer are related to oncogenic ERG deregulation

**DOI:** 10.1186/1471-2407-10-505

**Published:** 2010-09-22

**Authors:** Wolfgang A Schulz, Marc Ingenwerth, Carolle E Djuidje, Christiane Hader, Jörg Rahnenführer, Rainer Engers

**Affiliations:** 1 Department of Urology, Heinrich Heine University, Moorenstr. 5, 40225 Düsseldorf, Germany; 2 Department of Statistics, University of Dortmund, August-Schmidt-Straße 4, 44227 Dortmund, Germany; 3 Department of Pathology, Heinrich Heine University, Moorenstr. 5, 40225 Düsseldorf, Germany

## Abstract

**Background:**

The cortical cytoskeleton network connects the actin cytoskeleton to various membrane proteins, influencing cell adhesion, polarity, migration and response to extracellular signals. Previous studies have suggested changes in the expression of specific components in prostate cancer, especially of 4.1 proteins (encoded by *EPB41 *genes) which form nodes in this network.

**Methods:**

Expression of *EPB41L1*, *EPB41L2*, *EPB41L3 *(protein: 4.1B), *EPB41L4B *(EHM2), *EPB41L5*, *EPB49 *(dematin), *VIL2 *(ezrin), and *DLG1 *(summarized as „cortical cytoskeleton" genes) as well as *ERG *was measured by quantitative RT-PCR in a well-characterized set of 45 M0 prostate adenocarcinoma and 13 benign tissues. Hypermethylation of *EPB41L3 *and *GSTP1 *was compared in 93 cancer tissues by methylation-specific PCR. Expression of 4.1B was further studied by immunohistochemistry.

**Results:**

*EPB41L1 *and *EPB41L3 *were significantly downregulated and *EPB41L4B *was upregulated in cancer tissues. Low *EPB41L1 *or high *EPB41L4B *expression were associated with earlier biochemical recurrence. None of the other cortical cytoskeleton genes displayed expression changes, in particular *EPB49 *and *VIL2*, despite hints from previous studies. *EPB41L3 *downregulation was significantly associated with hypermethylation of its promoter and strongly correlated with *GSTP1 *hypermethylation. Protein 4.1B was detected most strongly in the basal cells of normal prostate epithelia. Its expression in carcinoma cells was similar to the weaker one in normal luminal cells. *EPB41L3 *downregulation and *EPB41L4B *upregulation were essentially restricted to the 22 cases with *ERG *overexpression. Expression changes in *EPB41L3 *and *EPB41L4B *closely paralleled those previously observed for the extracellular matrix genes *FBLN1 *and *SPOCK1*, respectively.

**Conclusions:**

Specific changes in the cortical cytoskeleton were observed during prostate cancer progression. They parallel changes in the expression of extracellular matrix components and all together appear to be associated with oncogenic ERG overexpression. We hypothesize that these alterations may contribute to the increased invasivity conferred to prostate cancer cells by ERG deregulation.

## Background

The progression of epithelial tumors to invasive carcinomas involves changes in cell polarity, adhesion and motility that permit the detachment of cancer cells from the epithelial layer, their invasion into adjacent tissue layers and eventually their spread throughout the body. These processes require reorganization of the cellular cytoskeleton and altered expression of proteins that connect it to the cell membrane as well as remodelling of the extracellular matrix including changes in the composition and processing of its constituents.

The 4.1 proteins, encoded by the *EPB41 *(erythrocyte protein band 4.1) genes, are components of the cortical cytoskeleton underlying the cell membrane [1,2]. The family of 4.1 proteins consists of the eponymous 4.1R protein first identified in erythrocytes (gene: *EPB41*), 4.1N (*EPB41L1*), 4.1G (*EPB41L2*), 4.1B (*EPB41L3*) as well as the less closely related members NBL4 (*EPB41L4A*), EHM2 (*EPB41L4B*) and EPB41L5 (*EPB41L5*). They form nodes in the cell cortex connecting further components of the cortical cytoskeleton like spectrins, actin and transmembrane adhesion proteins, receptors and transporters with each other. In this fashion 4.1 proteins contribute to the organization of cell polarity, adhesion and motility, and affect transport through the membrane and responses to growth factors.

The 4.1B protein is most strongly expressed in neurons and is enriched in the basal cells of certain epithelia [2,3]. In addition to spectrins and actin, known binding partners comprise the adhesion molecule CD44 that binds hyaluronic acid in the extracellular matrix [1] and the candidate tumor suppressor disc large 1/DLG1 [4]. The 4.1B protein is downregulated in several carcinomas, including prostate cancer [5], likely by deletion or promoter hypermethylation of the *EPB41L3 *gene promoter [6]. Mouse models of prostate cancer progression suggest that it acts as a metastasis suppressor [7]. In contrast, EHM2 is conspicuous in tumor cells with high migratory potential, such as metastatic melanoma and fibrosarcoma cells [8,9]. In prostate cancer, EHM2 has been reported to be overexpressed [6,10] and to diminish adhesion of prostate cancer cells to collagen [10]. The most recently discovered 4.1 family member, the product of the *EPB41L5 *gene (also called Limulus), regulates cell adhesion during development [11], but has not yet been investigated in the context of human cancer.

The 4.1 proteins are part of a larger protein family characterized by FERM domains, of which many have related functions. For instance, the FERM domain protein ezrin, encoded by the *VIL2 *gene, connects CD81 at the cell membrane to the actin cytoskeleton. Ezrin has been shown to mediate invasion of prostate cancer cells [12,13], but whether it is overexpressed in prostate cancer is not known. The more distantly related protein dematin, too, interacts with the actin cytoskeleton and growth factor receptors. It is encoded by the *EPB49 *gene on 8p21.1, a region frequently deleted in prostate cancer. Overexpression of dematin in PC3 prostate cancer cells changed their morphology towards a more epithelial phenotype [14], but no investigations of *EPB49 *expression in prostate cancer tissues have been published.

The fundamental reorganization of the cytoskeleton, its attachment to the cell membrane and the extracellular matrix during cancer progression are orchestrated by transcription factors that activate cellular programs for cell migration and invasion, which are physiologically employed during embryogenesis, wound healing and tissue regeneration [15,16]. In cancer, such transcription factors, like Snail/SNAI1, Slug/SNAI2 and ZEB1, often become deregulated and promote tumor progression.

In prostate carcinoma, transcription factors of the ETS family are prominent candidates for oncogenes driving this facet of tumor progression. Specific members of this protein family are activated towards oncogenes by chromosomal translocations [17] placing a structural ETS transcription factor gene under the control of an androgen-responsive promoter, resulting in its deregulation and overexpression. The most common translocation, found in 30-70% of all cases, creates a fusion gene placing the androgen-responsive promoter of *TMPRSS2 *in control of the *ERG *structural gene encoding an ETS family transcription factor. This genetic aberration results in the androgen-driven overexpression of intact or amino-terminally truncated ERG proteins in prostate epithelial cells. ERG oncoproteins influence tumor cell proliferation, but exert a more pronounced effect on migration and invasion through broad changes in gene expression [18-20]. In accord with a function in promoting tumor progression, *TMPRSS2-ERG *translocations are observed in a significantly lower fraction of high-grade prostate intraepithelial neoplasias, a non-invasive precursor stage, than in invasive carcinomas [21].

We have previously reported downregulation of *EPB41L3 *encoding protein band 4.1B and upregulation of *EPB41L4B *encoding EHM2, respectively, in prostate cancer [6], in accord with observations by other groups [7,10]. In the present study, we have investigated the expression of further members of the family as well as selected genes encoding related or interacting proteins such as disc large 1, ezrin and dematin, and the relation of the changes to activation of oncogenic ERG.

## Methods

### Tissues

High quality RNA was available from 13 benign prostate tissues and 45 cancer samples collected from patients undergoing radical prostatectomy for prostate carcinoma between 1997 and 2002 in our institution as described in a prior study [Schulz et al, 2007]. TNM classification was performed according to the rules of the International Union against Cancer from 2002. Twenty cancers were staged as pT2, 23 as pT3 and two as pT4. Twenty-six cancer specimens had a Gleason score of 7, 13 < 7 and 6 > 7. At the time of surgery, no distant metastases were detectable, but 11 patients had lymph node metastases. The patients' age ranged from 59 to 74 years. Follow-up data were available for all patients with a median follow-up time of 98 months. High quality DNA was available from 93 cancer tissues essentially encompassing the 45 specimens used for RNA analysis. Of these 93 cases, 44 were staged as pT2 and 49 as pT3 or pT4. Sixteen patients had lymph node metastases, but no distant metastases were detected. Each 27 carcinomas were assigned a Gleason score > 7 or <7 and 39 a score of 7. The study was approved by the ethics committee of the Heinrich Heine University medical faculty.

### DNA and RNA extraction

DNA and RNA were extracted from identical powdered tissues as described previously [22,23]. High molecular weight genomic DNA was isolated using the blood and cell culture DNA kit (Qiagen, Hilden, Germany). Total RNA was isolated using the RNeasy Mini Kit (Qiagen) following guanidinium/acid phenol/chloroform extraction (peqGOLD TriFast, peqLab, Erlangen, Germany). Quality of DNA and RNA was initially checked by spectrophotometry and subsequently by agarose gel or capillary electrophoresis, respectively. Only high quality DNA and RNA preparations were used in the present study.

### Reverse transcription and quantitative RT-PCR

cDNA was prepared using SuperscriptII (Invitrogen, Karlsruhe, Germany) according to the manufacturer's protocol with a mixture of random and oligo-dT primers. Quantitative real-time RT-PCR was performed on an ABI 7900 instrument using commercially available Taqman assays specific for the respective mRNAs (Applied Biosystems, Weiterstadt, Germany), namely HS 00938024-m1 (*DLG1*), HS 00385004_m1 (*EPB41L1*), HS 00154988_m1 (*EPB41L2*), HS 00202360_m1 (*EPB41L3*), HS 00603031_m1 (*EPB41L4B*), HS 01554437_m1 (*EPB41L5*), HS 00157387_m1 (*EPB49*), HS 00267195_m1 (*MKI67*), HS 0018574_m1 (*VIL2*). *TBP *(HS 00427620_m1) was used as a reference gene throughout. The assay for *ERG *was chosen to cover exons common to all oncogenic transcripts [19]. All reactions were performed with cDNA corresponding to 10 ng of RNA each in at least duplicates using 40 cycles of 15 s at 95°C and 60 s at 60°C after an initial 10 min denaturation at 95°C. Each run was standardized using a dilution series of cDNA from a strongly expressing cell line or normal tissue. Experimental variation for each sample was below 10%.

### DNA methylation analysis

DNA was bisulfite-treated using the EZ DNA Methylation Gold Kit (Zymo Research, Freiburg, Germany), Methylation-specific PCR for *GSTP1 *was performed as described [22] and accordingly for *EPB41L3 *using the primer pairs 5'-TTTGTGTATTGTTGTTGAGGAGTG-3' and 5'-CACAATCCCCCACTCCAAAAAACA-3' to detect unmethylated sequences or 5'-TTGCGTATCGTCGTCGTCGAGGACG-3' and 5'-CGCAATCCCCCACTCCGAAAAACG-3' to detect methylated sequences at 61°C and 64°C annealing temperature, respectively. Bisulfite sequencing of the *EPB41L1 *promoter was conducted by the method described previously [22] with the newly designed primers 5'-AGAGGAGATAGGTAGAGAGGA-3' and 5'-CTAAACCC(A/G)AAATTCCCCATATC-3' at 58°C.

### Immunohistochemistry

For immunohistochemistry paraffin sections of tissue microarrays comprising prostate cancer and corresponding benign prostatic tissue samples were treated with xylene and ethanol. Sections were rehydrated and endogenous peroxidase activity was eliminated by H_2_O_2_. After antigen retrieval using a citrate buffer in a pressure cooker, endogenous biotin was blocked. Slides were incubated with a mouse monoclonal antibody against 4.1B (Santa Cruz Biotechnology, dilution 1:10) overnight at 4°C. After a second incubation with a biotin-conjugated polyvalent antibody, slides were mixed with an avidin-biotin-peroxidase reagent (Scy Tek, Logan, USA). Reaction products were visualized by immersing slides in diaminobenzidine tetrachloride and finally counter-stained with haematoxilin. For negative controls, slides were subjected to the entire immunohistochemical procedure except for the fact that the primary antibody was omitted. Under these conditions no staining was observed.

### Statistical methods

All statistical calculations were performed with SPSS 18.0. For comparisons between groups Mann-Whitney U test was employed, correlations were evaluated by Spearman's rho, nominal data were evaluated by chi-square test and follow-up data by log-rank test.

## Results

### Expression of EPB41 and related genes in prostate cancer tissues

**Figure 1:**
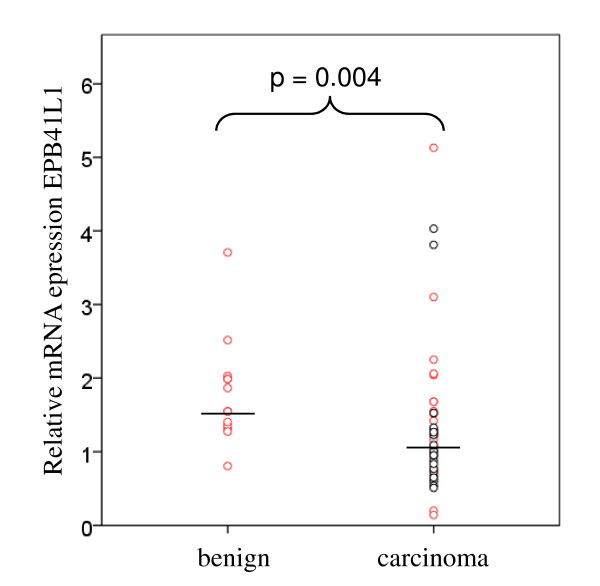
**Expression of *EPB41L1 *in prostate cancers**. Expression of *EPB41L1 *mRNA relative to *TBP *in prostate carcinoma and benign tissues. Black circles denote carcinoma specimens with ERG overexpression. Median values are indicated by horizontal lines.

Expression of *EPB41L1*, *EPB41L2*, *EPB41L3*, *EPB41L4B*, *EPB41L5*, *EPB49*, *VIL2*, and *DLG1 *(summarized as „cortical cytoskeleton" genes) was measured by quantitative RT-PCR in a well-characterized set of 45 M0 prostate adenocarcinoma tissues and 13 benign tissues from cancer-carrying prostates. As reported previously [6], *EPB41L3 *mRNA was highly significantly (Mann-Whitney p < 0.001) diminished and *EPB41L4B *increased (p = 0.002) in these cancer tissues. The expression of *EPB41L1 *was decreased moderately, but significantly in cancer tissues (Fig. 1). The expression of the five other genes remained unchanged on average (Additional file 1, Figure S1).

**Figure 2:**
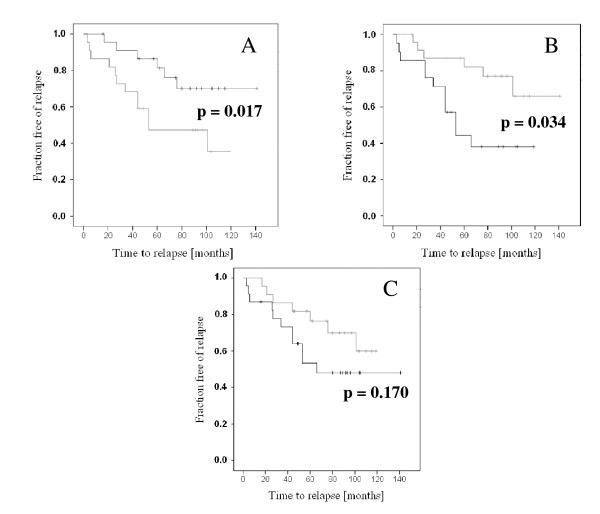
**Relation of *EPB41L3 *and *EPB41L4B *expression to prostate cancer recurrence**. Kaplan-Meyer analysis of biochemical recurrence in patients with below (lower line in B and C, upper line in A) or above (upper line in B and C, lower line in A) median expression of *EPB41L4B *(A), *EPB41L1 *(B) and *EPB41L3 *(C). The respective log rank p-values were 0.017, 0.034, and 0.170.

Within the cancer specimens, none of the genes showed a significant association of its expression level with tumor stage (pT2 vs. > pT2), Gleason score (< 7 vs. 7 vs. > 7) or lymph node metastasis. Below median expression of *EPB41L1 *was associated with significantly earlier biochemical recurrence, as was above median expression of *EPB41L4B *(Fig. 2). In contrast, decreased expression of *EPB41L3 *showed only a tendency towards earlier tumor relapse (Fig. 2).

### Expression of 4.1B protein in normal and cancerous prostate tissue

**Figure 3:**
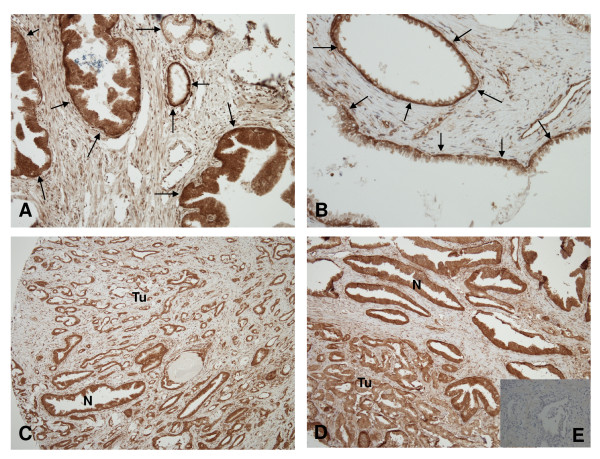
**Immunohistochemical analysis of protein 4.1B expression in prostate cancer and benign prostatic glands**. A and B: Heterogeneous expression of protein 4.1B in benign prostatic glands. Note stronger expression levels in basal cells (arrows) than in secretory epithelial cells. C-D: Comparison of 4.1B expression levels in tumor glands (TU) and corresponding benign prostatic glands (N): C and D: equivalent and slightly decreased, respectively, 4.1B expression in cancer cells compared to secretory cells of normal glands. Original magnifications: A, D, x200; B, x400; C, x100. E: negative control without primary antibody.

Immunohistochemical investigation (Fig. 3) revealed that in normal prostate glands, 4.1B expression was usually much stronger in basal cells than in secretory epithelial cells. Of note, although all glands stained positive, 4.1B expression was quite variable between individual glands (Fig. 3A, B). In cancerous glands, 4.1B protein remained detectable. In most cases staining intensity in tumor glands was similar to that of corresponding benign secretory epithelial cells within the same section (Fig. 3C). In some cases, 4.1B expression in carcinoma cells was decreased as compared to secretory epithelial cells of normal glands (Fig. 3D), but the opposite tendency was also observed in a few specimens.

### DNA methylation analysis of EPB41L3 and EPB41L1

Decreased expression of *EPB41L3 *was previously shown to be associated with methylation of its gene promoter in prostate cancer cell lines and a smaller series of prostate cancer tissues [6]. To more precisely measure its frequency and relationship to clinical parameters, the previously analyzed series was extended to 93 cancer specimens that were investigated by MS-PCR as described (a typical result is shown in Additional File 2, Figure S2). Of these, 61 (65%) cases displayed *EPB41L3 *hypermethylation. Hypermethylation was more frequent in higher stage cases, i.e. detectable in 25/44 (57%) of pT2 vs. 35/49 (71%) of higher stage specimens and 12/16 (75%) lymph-node positive vs. 49/78 (63%) lymph-node negative cancers, but the difference was not statistically significant (χ^2 ^> 0.05). Average expression of *EPB41L3 *mRNA was significantly lower in carcinomas with hypermethylation of the gene than in those without (1.79 ± 0.50 vs. 1.33 ± 0.61; p = 0.025). In the same tissue series, 71 (76%) carcinomas displayed hypermethylation of *GSTP1*. Hypermethylation of *EPB41L3 *was significantly (p = 0.002) more frequent in tumors with *GSTP1 *hypermethylation than in those without.

To investigate methylation as a potential cause of diminished *EPB41L1 *expression in cancer, 47 CpG sites around the transcriptional start site (from bp -208 to +157) were analyzed by bisulfite sequencing in the prostate cancer cell lines PC3 and LNCaP, in two prostate cancer tissues with lowered expression, one benign prostate sample and in leucocytes. None of them displayed any methylation, whereas an artificially methylated DNA yielded the expected fully methylated sequence (data not shown).

### Relation to changes in expression of other genes

Next, the expression of all cortical cytoskeleton genes in the cancer tissues was compared to that of *MKI67*, encoding the well established marker of cell proliferation in cancer, Ki67. Expression of *EPB41L4B *was positively (Spearman rho = 0.535, p < 0.001) and expression of *EPB41L3 *negatively (ς = - 0.411, p < 0.003) correlated with the proliferation marker.

**Figure 4:**
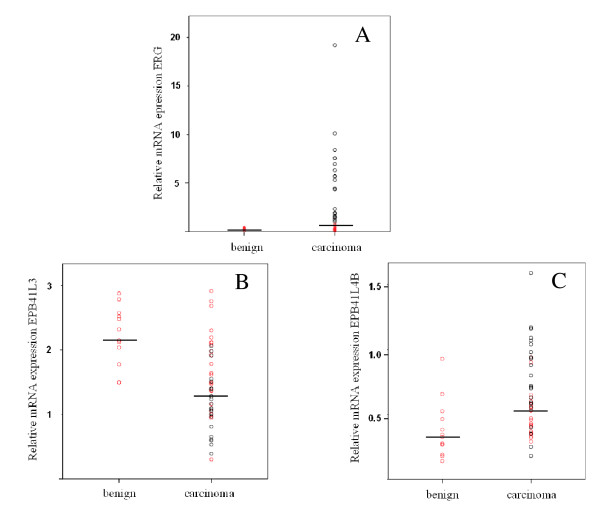
**Expression of *ERG *and its relation to *EPB41L3 *and *EPB41L4B *in prostate cancers**. Expression of *ERG*, *EPB41L3 *and *EPB41L4B *mRNAs relative to *TBP *in prostate carcinoma and benign tissues. Black circles denote carcinoma specimens with *ERG *overexpression. Median values are indicated by horizontal lines.

In various studies, approximately half of all prostate cancers have been observed to overexpress the ETS family gene *ERG*, predominantly as a consequence of different chromosomal translocations [24]. To cover all instances of ERG overexpression, we determined the expression of an mRNA segment common to all normal and oncogene transcripts by qRT-PCR and defined overexpression of *ERG *as a level exceeding twice that of the maximum of benign tissues. By that definition in the present series 22 of 45 cancers overexpressed ERG, by up to two orders of magnitude (Fig. 4A). Across all cases, *ERG *expression was significantly different between cancer and benign tissues (Mann-Whitney-U p = 0.029).

Subsequently, expression of the cortical cytoskeleton genes was compared between the groups with elevated (ERG-high) and close to normal (ERG-low) *ERG *expression (marked in black and red, respectively in Figs. 1 and 4). Expression of *EPB41L4B *was significantly stronger (p = 0.01) and *EPB41L3 *significantly lower (Mann-Whitney U p = 0.01) in ERG-high specimens (Fig. 4B, C). It is evident from the figure that the majority of ERG-high cancers displayed decreased expression of *EPB41L3 *and increased expression of *EPB41L4B*, whereas most of the ERG-low carcinomas had expression levels of the two genes within the range of benign tissues. In a similar fashion, *EPB41L1 *expression was on average lower in ERG-high than ERG-low cancers (Fig. 1), but in this case the difference was not statistically significant. None of the other cortical cytoskeleton genes differed significantly with respect to its expression between ERG-high and ERG-low cancer tissues.

When comparing these results to previous data from our group, we noted a close correlation (Spearman rho = 0.807, p < 0.001) between the expression of *EPB41L4B *and *SPOCK1*, the gene encoding the extracellular matrix protein testican 1. *SPOCK1 *expression is upregulated in prostate cancer and associated with diminished expression of *FBLN1 *encoding the basement membrane component fibulin 1 [25]. Accordingly, expression of *FBLN1 *was correlated inversely with that of *EPB41L4B *(rho = - 0.739, p < 0.001), but correlated well positively with that of *EPB41L3 *(rho = 0.684, p < 0.001). Moreover, *SPOCK1 *expression was significantly elevated (p = 0.002) in ERG-high cancers and that of *FBLN1 *was significantly diminished (p = 0.047). The expression of other cortical cytoskeleton genes was not related to either *SPOCK1 *or *FBLN1 *expression.

## Discussion

Previous studies have concurrently reported changes in the expression of *EPB41L3*/4.1B and *EPB41L4B*/EHM2 in many prostate cancers. The other members of the 4.1 family had not been studied yet in prostate cancer, but previous investigations have indicated potential functions for the related proteins ezrin and dematin [12-14,26,27]. Despite these hints, we did not observe significant changes in ezrin/*VIL2 *or dematin/*EPB49 *mRNA expression in prostate cancer compared to benign tissues. Instead, the present investigation indicated that the expression changes of *EPB41L3 *and *EPB41L4B *are quite specific among cortical cytoskeleton genes, with the exception of *EPB41L1*. Our findings therefore call for a detailed investigation of 4.1N expression and function in prostatic cells in future studies.

Investigation of *EPB41L3 *methylation in a larger series of samples confirmed the presumed association between its downregulation and hypermethylation. *EPB41L3 *hypermethylation tended to be more frequent in higher stage tumors and downregulation of gene expression tended to be associated with earlier recurrence. The two changes did not correlate significantly with the same clinical indicators of tumor progression, which may be due the limited number of samples studied, since the tendencies were the same. In any case, these findings concur with the idea from functional studies in mouse and cell line models that 4.1B may act as a metastasis suppressor in prostate cancer [7]. Likewise, although we did not observe significant associations of *EPB41L4B *expression with cancer stage or grade, increased mRNA expression was associated with earlier recurrence, in accord with the immunohistochemical study of Wang et al. [10].

Moreover, the extended *EPB41L3 *methylation analysis allowed a comparison with the well-studied hypermethylation of *GSTP1*. Hypermethylation of *GSTP1 *can be detected in a significant fraction of HG-PINs [28], but more consistently after progression towards invasive carcinomas. It is thought to occur as part of an „epigenetic catastrophe" [29] that involves hypermethylation of further genes, e.g. *APC*, *RARB2 *and *RASSF1A*. In our tissue series, accordingly, cases with hypermethylation of *GSTP1 *have a very high likelihood of displaying hypermethylation of these genes as well [22]. *EPB41L3 *hypermethylation appears to occur slightly less frequently and was notably essentially restricted to cases with *GSTP1 *hypermethylation. A straightforward interpretation of this finding is that *EPB41L3 *hypermethylation occurs in many prostate cancers in conjunction with or following the hypermethylation of *GSTP1*.

In the present study, we observed a good correlation between the presence of *EPB41L3 *and *EPB41L4B *expression changes on one hand and *ERG *overexpression on the other hand. Simply stated, carcinoma tissues without *ERG *overexpression showed no differences in expression of the 4.1 genes towards normal tissues (Fig. 4). Our data therefore suggest that the changes in the expression of the two cortical cytoskeleton genes might be a consequence of ERG overexpression. This hypothesis fits with the presumed sequence of events during prostate cancer development. Fusion genes causing ERG overexpression can be detected in some pre-neoplastic HG-PINs, but more consistently in invasive carcinomas [21] supporting a role of ERG in promoting prostate cancer invasion and progression. As mentioned above, this transition is also associated with consistent hypermethylation of *GSTP1*, which may coincide with or precede hypermethylation of *EPB41L3*.

A relationship between ERG and the cortical cytoskeleton proteins is also plausible on functional grounds. Overexpression of ERG is thought to result in increased proliferation, invasiveness and motility of prostate cancer cells [18-20,30], whereas protein 4.1B has been shown to oppose invasiveness and metastasis of prostate cancer cells [5,7] and EHM2 to modulate adhesion of prostate cancer cells [10]. Our data invite the interpretation that the changes in *EPB41 *gene expression are part of a presumed invasion program directed by ERG and serve to implement the necessary changes in the cortical cytoskeleton. Whether cortical cytoskeleton genes are indeed - directly or indirectly - regulated by ERG, must now be investigated in cell line and animal models.

Along the same line of argument, we have previously observed changes in the expression of ECM genes encoding fibulin 1 (*FBLN1*) and testican 1 (*SPOCK1*). The causes of these changes are so far unclear, since we did not observe altered gene copy numbers in the majority of cases or obtain evidence for *FBLN1 *hypermethylation [25]. We have now become aware that upregulation of *SPOCK1 *parallels that of *EPB41L4B *and downregulation of *FBLN1 *parallels that of *EPB41L3*. Accordingly, the changes in the expression of the ECM genes were also associated with *ERG *overexpression. They may therefore represent changes in the composition of the extracellular matrix brought about as a consequence of ERG oncogenic activation. The observation that EHM2 influences cell adhesion to basement membrane collagen is intriguing in that respect [10]. Of note, the strikingly close correlation between *EPB41L4B *and *SPOCK1 *expression in the tissue samples may be enhanced by their common regulation by androgens [10,31]. It remains to be determined whether the ECM genes are indeed ERG targets.

In this study, we also report the first immunohistochemical investigation of 4.1B protein in prostatic tissues. Not unexpectedly [2,3], we observed the most prominent expression in the basal cells of the prostatic glands, while luminal cells contained less protein. Loss of the basal cell population is a well-established characteristic of prostate cancers. The downregulation of *EPB41L3 *expression may therefore largely reflect the loss of basal cells during prostate cancer development. Basal cells in the prostate epithelium express *GSTP1*, in contrast to luminal cells [32]. Accordingly, loss of expression and hypermethylation of *GSTP1 *may be related to the loss of the basal cell compartment [33]. Consequently, it is tempting to speculate that the hypermethylation of *EPB41L3 *in prostate cancer, too, might be associated with the loss of the basal cell compartment. Unlike GSTP1, however, 4.1B protein is expressed also in luminal cells of normal glands and the expression in cancerous prostatic glands remained overall comparable to that in the luminal cells, despite the presence of significant hypermethylation in a majority of the cases. Indeed, in prostate cancer cell lines with pronounced hypermethylation of the *EPB41L3 *gene and low levels of mRNA [6], a small amount of 4.1B protein remains detectable by blot techniques (Schulz et al., unpublished results). The residual expression of the gene in cancer cells could be due to a low level of transcription despite hypermethylation of the main promoter or from an alternative weaker promoter suspected in the gene [34].

## Conclusions

Our findings suggest that highly specific changes in the cortical cytoskeleton occur during the progression of prostate cancers which parallel changes in the expression of extracellular matrix genes. Since these changes are found especially in cases with ERG overexpression, we hypothesize that they may constitute a part of the invasion program induced by oncogenic ERG.

## Competing interests

The authors declare that they have no competing interests.

## Authors' contributions

WAS designed the study. ECD and CH performed RNA and DNA analyses, RE performed and evaluated immunohistochemistry. MI and JR conducted statistical analyses. WAS, MI, JR and RE interpreted the data. WAS, MI and RE wrote the manuscript. All authors have given final approval to the manuscript.

## Pre-publication history

The pre-publication history for this paper can be accessed here:

http://www.biomedcentral.com/1471-2407/10/505/prepub

## Supplementary Material

Additional file 1 **Expression of EPB41L2, EPB41L5, EPB49, VIL2, and  DLG1 mRNA in prostate tissues.** Box plot representation of expression of the indicated genes relative to TBP in prostate cancer and benign tissues as measured by qRT-PCR. Click here for file

Additional file 2 **MS-PCR for EPB41L3 methylation.** Photograph of ethidium-bromide stained agarose gel with PCR products from bisulfite-treated DNA from numbered tissue samples, leukocytes (Leu) as unmethylated control, Du145 (Du) as methylated control, or water (W), using primers specific for the unmethylated (U) or methylated (M) EPB41L3 promoter. The last lane (MW) contained the size marker. Click here for file
