# Supplementary figures and images for: Changes in cortical cytoskeletal and extracellular matrix gene expression in prostate cancer are related to oncogenic ERG deregulation
Source: BMC Cancer. 2010 Sep 22;10:505. doi: 10.1186/1471-2407-10-505 (PMC2955608; doi:10.1186/1471-2407-10-505)

## Slide 1
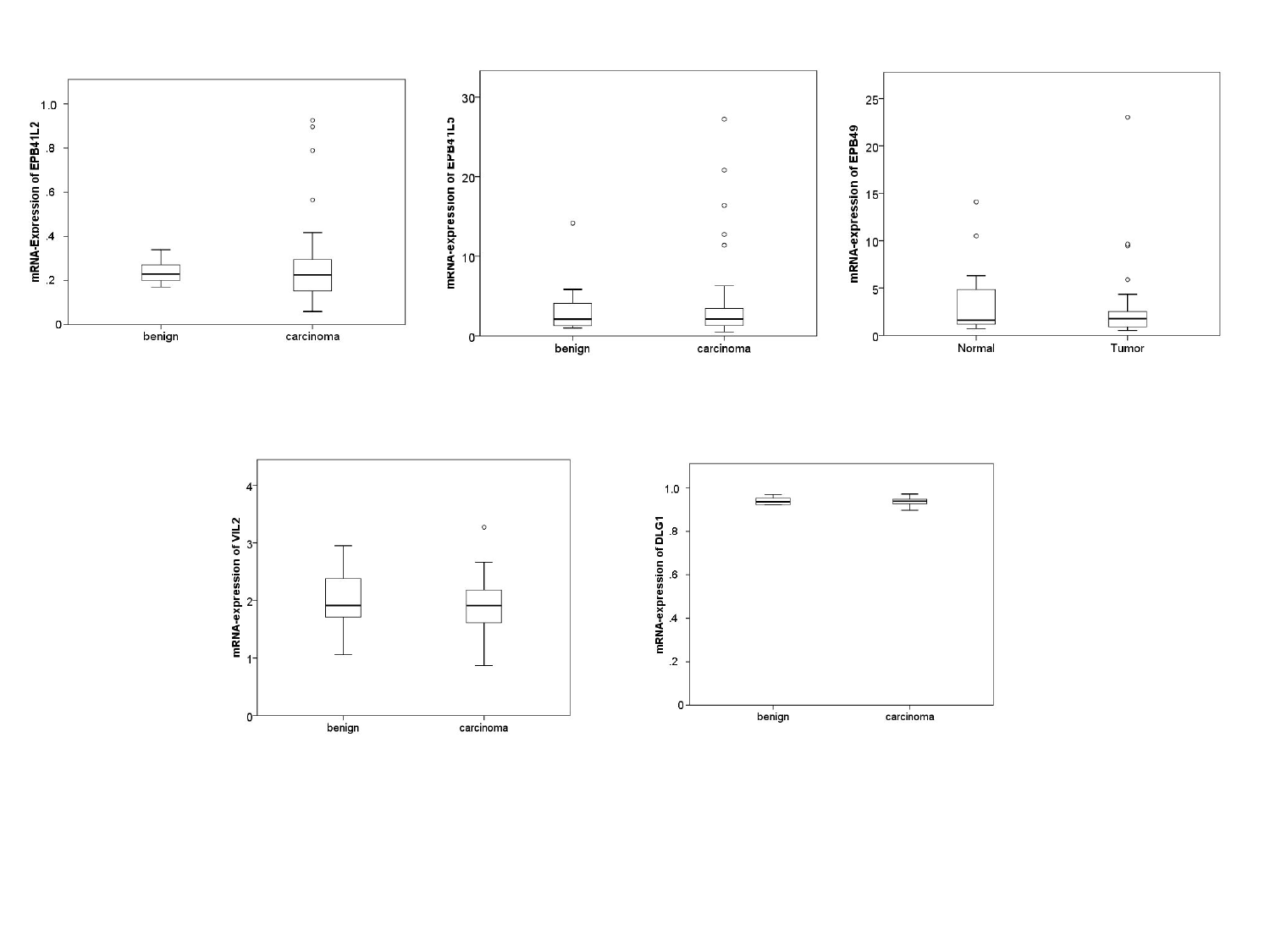

Supplement: Additional file 1 — Expression of EPB41L2, EPB41L5, EPB49, VIL2, and DLG1 mRNA in prostate tissues. Box plot representation of expression of the indicated genes relative to TBP in prostate cancer and benign tissues as measured by qRT-PCR. [file 1471-2407-10-505-S1.PPT]

## Slide 1
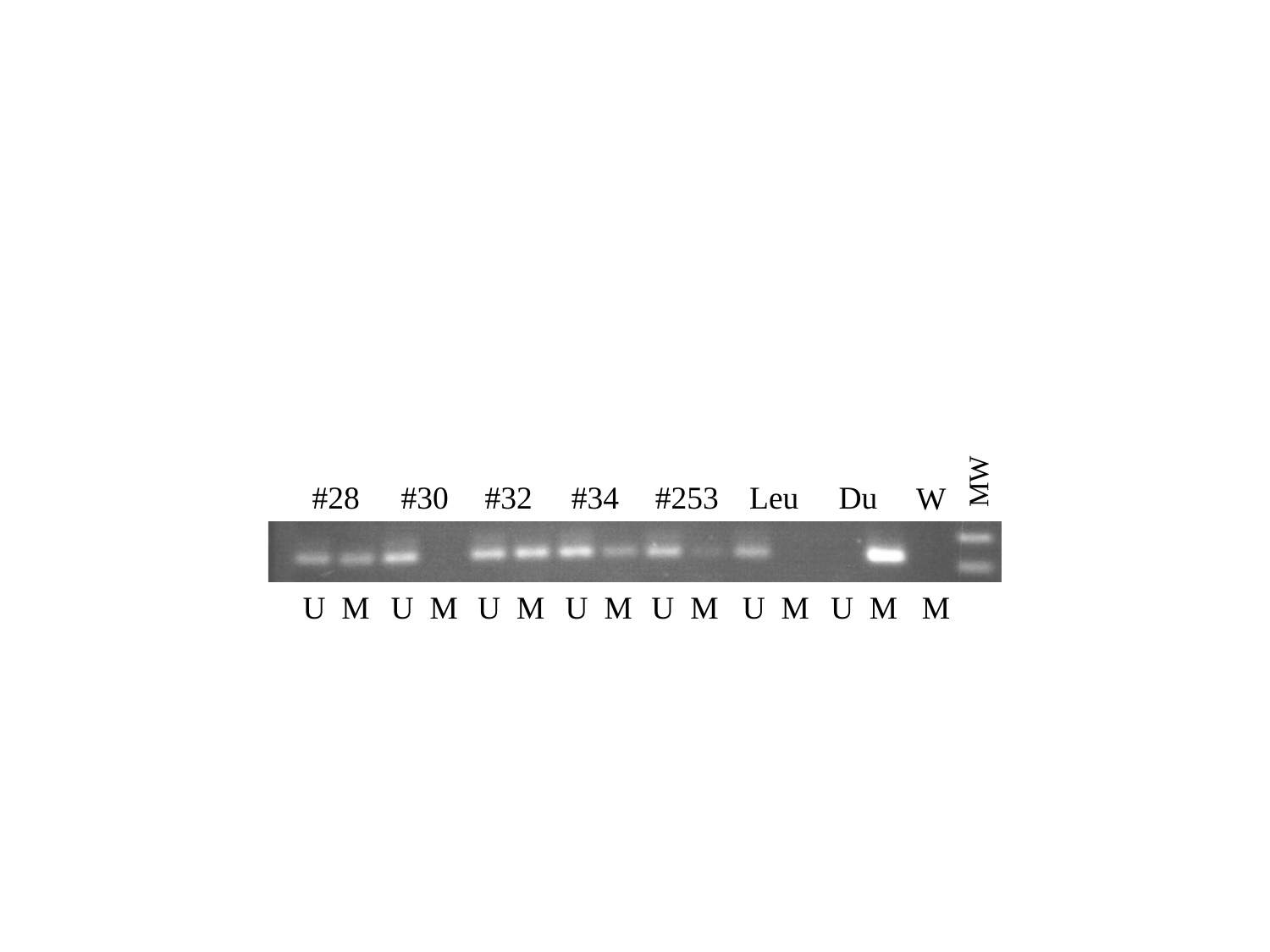

MW
#28
#30
#32
#34
#253
Leu
Du
W
U M
U M
U M
U M
U M
U M
U M
M

Supplement: Additional file 2 — MS-PCR for EPB41L3 methylation. Photograph of ethidium-bromide stained agarose gel with PCR products from bisulfite-treated DNA from numbered tissue samples, leukocytes (Leu) as unmethylated control, Du145 (Du) as methylated control, or water (W), using primers specific for the unmethylated (U) or methylated (M) EPB41L3 promoter. The last lane (MW) contained the size marker. [file 1471-2407-10-505-S2.PPT]
